# Weight Loss and Mortality in Overweight and Obese Cancer Survivors: A Systematic Review

**DOI:** 10.1371/journal.pone.0169173

**Published:** 2017-01-06

**Authors:** Sarah E. Jackson, Malgorzata Heinrich, Rebecca J. Beeken, Jane Wardle

**Affiliations:** Health Behaviour Research Centre, Department of Epidemiology and Public Health, University College London, London, WC1E 6BT, United Kingdom; University of Chieti, ITALY

## Abstract

**Background:**

Excess adiposity is a risk factor for poorer cancer survival, but there is uncertainty over whether losing weight reduces the risk. We conducted a critical review of the literature examining weight loss and mortality in overweight or obese cancer survivors.

**Methods:**

We systematically searched PubMed and EMBASE for articles reporting associations between weight loss and mortality (cancer-specific or all-cause) in overweight/obese patients with obesity-related cancers. Where available, data from the same studies on non-overweight patients were compared.

**Results:**

Five articles describing observational studies in breast cancer survivors were included. Four studies reported a positive association between weight loss and mortality in overweight/obese survivors, and the remaining study observed no significant association. Results were similar for non-overweight survivors. Quality assessment indicated high risk of bias across studies.

**Conclusions:**

There is currently a lack of observational evidence that weight loss improves survival for overweight and obese cancer survivors. However, the potential for bias in these studies is considerable and the results likely reflect the consequences of disease-related rather than intentional weight loss. There is a need for stronger study designs, incorporating measures of intentionality of weight loss, and extended to other cancers.

## Introduction

Overweight and obesity are highly prevalent [1] and associated with increased risk of a number of the most common cancers [2–4]. There is convincing evidence that having a higher body mass index (BMI) increases the risk of developing cancers of the uterus, gallbladder, kidney, colon, cervix, and breast (in postmenopausal women) [3,4]. Overweight and obese individuals are also probably at higher risk of liver cancer, and some evidence suggests they may also be at increased risk of cancers of the oesophagus, rectum, and ovary, as well as leukaemia, non-Hodgkin lymphoma, and multiple myeloma [3,4]. A recent study estimated that 3.6% of cancer cases diagnosed worldwide in 2012 were attributable to high BMI [5]. As such, many cancer survivors are overweight or obese at the time of diagnosis.

Advances in cancer screening and treatment mean that cancer survivors are living longer [6] and they are seeking information about how lifestyle factors may influence their prognosis [7–9]. For those who are overweight or obese, the potential implications of losing weight are particularly relevant. In the general population, a desire to weigh less is ubiquitous among overweight and obese individuals, and a substantial proportion report trying to lose weight [10–14]. Carrying excess weight has been identified as a risk factor for recurrence, second primary cancers, reduced treatment effectiveness, treatment-related complications, and mortality [15–27], and consequently a cancer diagnosis may serve as an added motivation to lose weight.

There has been relatively little intervention research into the effect of weight loss on cancer outcomes. Two large trials of dietary change in breast cancer survivors–the Women’s Intervention Nutrition Study (WINS) [28] and the Women’s Healthy Eating and Lifestyle (WHEL) study [29]–produced findings suggestive of a favourable effect of weight loss on recurrence. Both studies achieved positive changes in diet in the intervention group, but intervention participants in WINS also lost weight. Analyses of cancer outcomes found lower cancer recurrence rates in the intervention group in WINS. Smaller randomised controlled trials (RCTs) examining associations between weight loss and cancer-related biomarkers in overweight and obese survivors have provided evidence consistent with benefits on progression and recurrence [30–33], and further research is underway to explore these changes in more detail [34–40]. However, although these studies are supportive of the idea that weight loss could improve cancer outcomes, none has yet directly examined whether losing weight confers a survival advantage.

In this article we systematically review the available evidence on the relationship between weight loss and mortality in overweight and obese cancer survivors. Our aim was to establish whether weight loss conferred a mortality advantage for overweight and obese cancer survivors. For comparison, we included findings on the association between weight loss and mortality in non-overweight cancer survivors where it was reported within the same studies. Based on a critical review of the existing literature, gaps are identified and recommendations made for future directions in research and practice.

## Method

### Search strategy

The review methodology was developed in keeping with PRISMA guidelines for systematic reviews S1 Table. We systematically searched, with no language restrictions, PubMed and EMBASE (from their commencements through May 2014, and later updated to April 2016) for studies of the association between weight loss and mortality in overweight and obese cancer survivors. We intersected terms related to weight loss (weight loss*, weight change, weight reduction), mortality (mortality, survival, death), weight status (overweight, obese, obesity, BMI), and presence of cancer (cancer, malignan*, neoplasm*, carcinoma, tumour, tumor). All fields were searched and results were filtered to include only studies of adult human populations. We supplemented the electronic searches with manual searches of the reference lists and ‘cited by’ lists of the selected articles and relevant reviews [41–53]. We looked for both clinical trials and observational studies; the former are important for establishing a causal relationship between weight loss and mortality, while the latter are useful for examining associations in more representative samples of patients.

### Study selection

We followed PRISMA guidelines on reporting items for systematic reviews [54]. The eligibility of each article was assessed independently by two investigators (SEJ and MH). We included studies if they reported the association between weight loss and either cancer-specific or all-cause mortality (or survival) in overweight or obese cancer survivors. This included analyses of solely overweight (BMI 25–29.9), obese (BMI ≥30), or overweight and obese (BMI ≥25) patients, or analyses of patient groups across the whole weight spectrum that reported the association between weight loss and mortality stratified by weight status. We included studies in which height and weight (to calculate BMI) had been self-reported, as well as studies where they had been objectively measured.

In view of the limited number of studies available for review, we placed no restrictions on the timing of the baseline weight measurement (i.e. before or shortly after diagnosis) as long as the follow-up weight measurement was at least 12 months after diagnosis. The rationale for the choice of the post-diagnosis follow-up point was to allow reasonable time for the acute effects of treatment to have subsided.

Studies that examined cancer mortality in samples that were not limited to cancer survivors and included healthy individuals or patients without cancer were excluded. We also excluded studies of patients undergoing palliative treatment, studies that used recurrence-free survival as their outcome (and did not also report overall/cancer-specific survival), and studies that reported the interaction between weight loss and weight status but did not report any stratified results.

Finally, because it is reasonable to consider that intentional weight loss would affect outcomes in cancers with an aetiological link with obesity (e.g. endometrial or breast), but less plausible for non-obesity-related cancers (e.g. nasopharyngeal), we also excluded studies restricted to cancer types not convincingly associated with excess weight, as defined by the World Cancer Research Fund [2] and results of Bhaskaran et al.’s 2014 *Lancet* paper [4]. Although some reviews provide suggestive evidence of an association between obesity and poorer survival from cancer (e.g., see [24,26]), we do not believe it to be sufficient to warrant inclusion of cancer types that do not have an aetiological relationship with obesity. In a report by the World Cancer Research Fund on breast cancer survivorship, it was concluded that the evidence that greater body fatness after diagnosis increases risk of mortality is limited (i.e. not convincing or probable) [55]. A meta-analysis in prostate cancer survivors [24] raised substantial questions about the observed association between BMI and survival, with the authors suggesting it could be due to delayed diagnosis, more advanced stage at diagnosis, or treatment difficulties, which would not be ameliorated by weight loss. As such, we cannot know whether the poorer survival is directly attributable to excess weight or other factors. The results of that review were also limited by high heterogeneity across studies and various issues relating to missing data and failure to adjust for important confounders [24]. The authors highlighted the need for studies of biomarkers and genetic markers related to adiposity and energy metabolism to provide biological plausibility for a causal role of obesity in poorer cancer survival. Taking all of these factors into account, we did not feel that the evidence for an association between obesity and poor survival in non-aetiologically-linked cancers was convincing enough for studies in these cancer types to be included in our review.

Where we identified multiple records reporting the same analyses (e.g. a conference abstract and a full publication) we included only the most recent article. Non-English records retrieved were run through online translation programs to allow basic screening of the title and abstract; none were identified as relevant for full-text consideration.

### Data extraction

The following information was extracted independently from the included articles by two investigators (SEJ and MH): study design, sample size and description, type of disease, disease stage, length of follow-up, definition of weight loss, and the timing of weight assessments relative to diagnosis. We also extracted all-cause and cancer-specific mortality risk estimates and 95% confidence intervals (CI) associated with weight loss in overweight/obese survivors. Where available, the equivalent risk estimates for non-overweight cancer survivors (BMI <25) were also extracted to facilitate comparison. Where several models with different degrees of adjustment for potential confounders were reported, we extracted the maximally adjusted risk estimates. Where mortality was reported at several time points, we extracted data at the latest time point to give the longest possible follow-up period. We attempted to obtain additional information on each study as needed by contacting the lead or corresponding author of each study.

### Quality assessment

In the absence of an internationally accepted quality assessment tool for observational studies, we developed a six-item evaluation framework to assess methodological quality. Specifically, we sought to explore each study’s risk of bias, defined by the STROBE reporting recommendations as “a systematic deviation of a study's result from a true value” that is typically “introduced during the design or implementation of a study and cannot be remedied later” [56]. We identified six potential biases relevant to our study question (selection bias, treatment allocation bias, immortal time bias, additional treatment bias, adherence bias, and survival time bias/competing risk). We adopted a ‘signalling question’ and derived categories of high and low risk of bias for each one (S2 Table). A diagram was constructed for the included studies to illustrate contrasting biases.

## Results

### Search results

**Fig 1:**
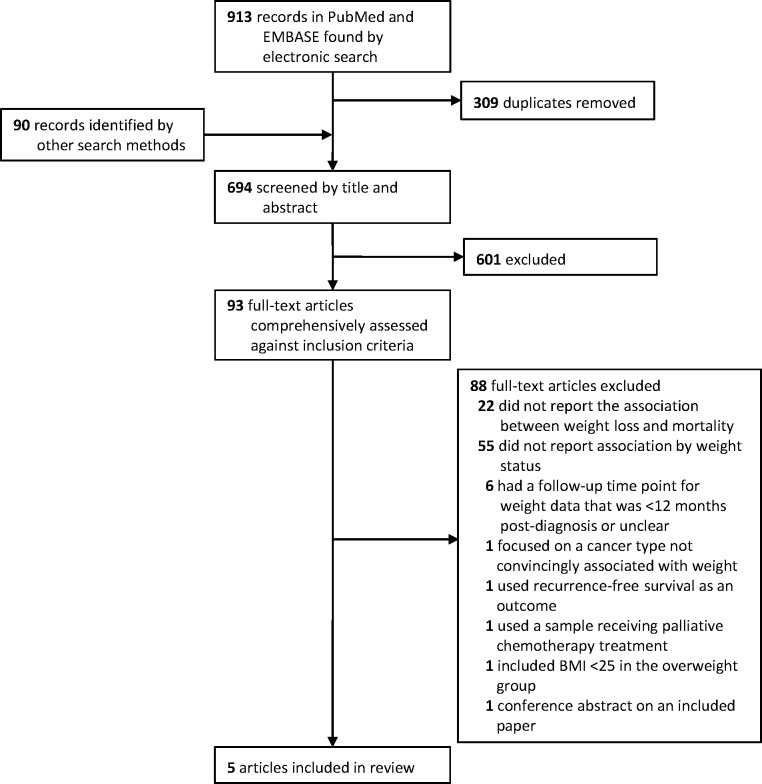
Flow diagram of search strategy and study selection.

In total, 694 unique articles were identified (604 by the electronic searches and 90 from other sources), which were reduced to 93 potentially eligible studies after screening the titles and abstracts according to our inclusion and exclusion criteria (Fig 1). The majority of excluded articles either did not focus on cancer or were not restricted to samples of cancer survivors. After a careful assessment of the remaining articles, we excluded those that did not report the association between weight loss and mortality, or did not present these data stratified by weight status; those that focused on recurrence-free survival as an outcome; involved patients receiving palliative treatment; had a follow-up time point for weight data that was less than 12 months post-diagnosis or was unclear; focused on cancers not convincingly associated with weight; included individuals with a BMI <25 in the ‘overweight’ group; or were conference abstracts of included papers. After these exclusions, five articles remained for the final synthesis [57–61].

### Overview of included studies

All the articles included in our review describe observational studies published in English between 2005 and 2012, with data collected between 1976 and 2008. No relevant clinical trials were identified. Table 1 summarises the characteristics of the five included studies.

**Table 1:** Characteristics of studies investigating the association between weight loss and mortality in overweight and obese cancer survivors.

Author (year)	Country (cohort acronym)	Design	Population	*n*	*n* non-overweight/ overweight	Mean age in years	Assessment of weight and definition of weight loss	Definition of weight loss	Referent category	Mortality outcomes	Median follow-up in months	Covariates	Risk estimates: HR/RR (95% CI)
Bradshaw (2012)	USA (LIBCSP)	Observational	Patients with a first primary in situ or invasive breast cancer (stage not specified)	1436	664 / 772	59	Participants reported their weight 1 year before diagnosis, at diagnosis, 1 year after diagnosis, and at follow-up (approx. 5 years after diagnosis).	Weight loss ≥5% post-diagnosis	Weight stable ±5%	All-cause Cancer-specific	106	Age at diagnosis, pre-diagnosis BMI, pre-diagnosis adult weight gain, tumour size, oestrogen and progesterone receptor status, chemotherapy	*All-cause mortality*: Overweight: 4.75 (2.80–8.41); Non-overweight: 7.43 (4.09–14.1). *Cancer-specific mortality*: Overweight: 7.84 (3.36–21.2); Non-overweight: 7.98 (3.51–19.0)
Caan (2012)	USA (LACE, WHEL, NHS) China (SBCSS)	Observational	Patients with invasive breast cancer (stage I-IV)	USA 8429; China 4486	USA 3962 / 4392; China 2984 / 1457	USA 59; China 54	Participants reported their weight 1 year before diagnosis and 18–48 months after diagnosis (post-diagnosis weight was measured in the WHEL cohort).	Weight loss of 5–10% (moderate) or ≥10% (large) from pre- to post-diagnosis	Weight stable ±5%	All-cause	97	Age at diagnosis, race, menopausal status, stage, positive nodes, oestrogen and progesterone receptor status, radiotherapy, chemotherapy, smoking	*USA (moderate weight loss)*: Overweight: 1.05 (0.82–1.33); Non-overweight: 1.59 (1.17–2.16). *USA (large weight loss)*: Overweight: 1.40 (1.09–1.81); Non-overweight: 1.74 (1.16–2.60). *China (moderate weight loss)*: Overweight: 0.99 (0.57–1.72); Non-overweight: 2.62 (1.58–4.36). *China (large weight loss)*: Overweight: 1.74 (1.07–2.83); Non-overweight: 4.08 (2.35–7.11)
Chen (2010)	China (SBCSS)	Observational	Patients with breast cancer (stage 0-IV)	5042	3378 / 1664	54	Participants reported their weight 1 year before diagnosis and at diagnosis, and weight was measured at 6 and 18 months after diagnosis.	Weight loss >1kg from diagnosis to 18 months post-diagnosis	Weight stable ±1kg	All-cause	46	Age at diagnosis, education, income, marital status, comorbidity, exercise participation, intake of meats, cruciferous vegetables, and soy protein, time interval from diagnosis to study enrolment, menopausal status, menopausal symptoms, chemotherapy, type of surgery, radiotherapy, immunotherapy, tamoxifen use, oestrogen and progesterone receptor status, tumour-node metastasis stage	Overweight: 2.00 (1.13–3.53). Non-overweight: 2.69 (1.60–4.52)
Caan (2008)	USA (LACE)	Observational	Patients with invasive breast cancer (stage I-IIIA)	2288	1283* / 409 (*BMI <30 –includes overweight category so results not included in review)	58	Participants reported their weight 1 year before diagnosis and at study entry (average of 5 years after diagnosis).	Weight loss of 5–10% (moderate) or ≥10% (large) from pre- to post-diagnosis	Weight stable ±5%	All-cause	60	Age at diagnosis, stage, tamoxifen use, chemotherapy, radiotherapy, number of positive nodes, oestrogen and progesterone receptor status, smoking history, physical activity	*Moderate weight loss*: Overweight: 0.4 (0.1–1.5). *Large weight loss*: Overweight: 2.8 (1.4–5.6)
Kroenke (2005)	USA (NHS)	Observational	Patients with invasive breast cancer without in situ disease or metastatic cancer at diagnosis (stage I-III)	5204	2719 / 2485	59	Participants reported their weight in biennial surveys. Pre-diagnosis weight was defined as the biennial survey prior and most recent to diagnosis (if missing, previous biennial survey) and post-diagnosis weight was defined as the survey after diagnosis if reported weight was ≥12 months after diagnosis, to allow for completion of treatment (if missing, next biennial survey).	A reduction in BMI >0.5kg/m^2^ from pre- to post-diagnosis	Weight stable ±0.5kg/m^2^	Cancer-specific	108	Age, pre-diagnosis BMI, oral contraceptive use, parity and age at birth, menopausal status, age at menopause, use of hormone replacement therapy, protein intake, chemotherapy and tamoxifen use	Overweight: 0.81 (0.57–1.15); Non-overweight: 1.41 (0.95–2.09)

LIBCSP: Long Island Breast Cancer Study Project. LACE: Life After Cancer Epidemiology. WHEL: Women’s Healthy Eating and Living Study. NHS: Nurses’ Health Study. SBCSS: Shanghai Breast Cancer Survival Study.

#### Study populations

All studies focused on survivors of breast cancer. Three studies drew their samples from the USA [57,58,61], one was conducted in China [60], and one was cross-national, with samples from the USA and China [59]. In the latter study, the Chinese sample was drawn from the same cohort as was used in another included study (the Shanghai Breast Cancer Survival Study), and the USA sample was drawn from three different cohorts, two of which were used in other included studies (Life After Cancer Epidemiology and the Nurses’ Health Study) and one of which was not reported on in any other included article (the Women’s Healthy Eating and Living Study). While the specific inclusion criteria varied by study, there was considerable overlap between these study populations. Most cohorts in the included studies specifically recruited breast cancer survivors; the exception was the Nurses’ Health Study, a large prospective cohort study of female nurses.

The mean age of participants ranged from 54 to 59 years, and the total sample size ranged from 1436 to 12915 cancer survivors. Each study grouped participants by weight status based on BMI; the majority used a threshold of 25kg/m^2^ to distinguish overweight or obese (BMI ≥25) versus underweight or healthy weight (<25), but one classified participants as obese (BMI ≥30) or non-obese (BMI <30) [58]. The number of cancer survivors in the overweight/obese category ranged from 409 to 5849 (18% to 54% of the total study sample) across studies.

#### Assessment of weight change

The assessment of weight and definition of weight loss varied across studies. The majority relied on retrospective self-reports of weight at both time points, but objective weight measurements were obtained at follow-up in two cohorts [59,60]. Three studies classified weight change according to the percentage change in body weight and used a cut-off of 5% to indicate weight loss [57–59]. One study used weight change (>1kg) to define weight loss [60], and one used BMI change (>0.5kg/m^2^) [61]. Two studies used multiple categories of weight loss, defining a loss of 5–10% of initial body weight as ‘moderate’ weight loss, and ≥10% as ‘large’ weight loss [58,59]. None distinguished between intentional and unintentional weight loss. In all studies, weight loss was compared to a weight stable referent category, defined as weight change <5% [57–59], <1kg [60], or a BMI change <0.5kg/m^2^ [61].

#### Mortality end-points

Four studies reported all-cause mortality [57–60] and two reported cancer-specific mortality [57,61]. Just one study reported associations with both all-cause and cancer-specific mortality [57]. The mean follow-up period was seven years (range 46–108 months).

### Quality assessment

**Fig 2:**
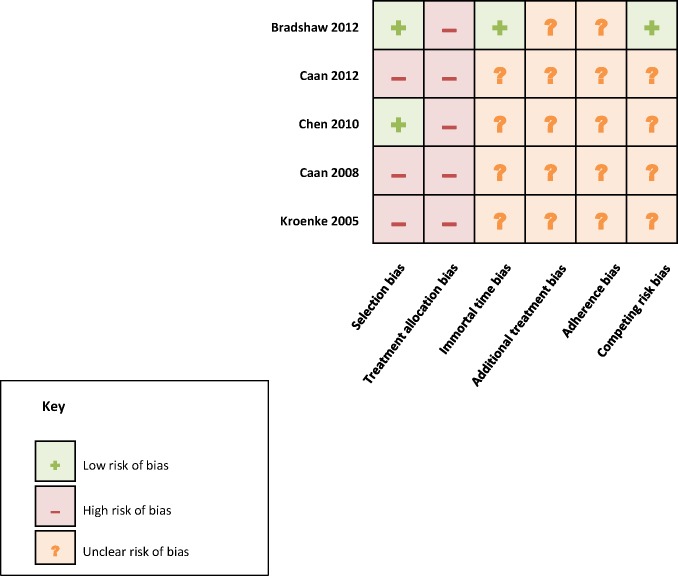
Assessment of studies’ risk of bias.

We formally assessed each study against six domains of potential for bias and the results are shown in Fig 2. Two studies were considered to be broadly representative of the patient population, with one recruiting participants through hospitals [57] and the other through a cancer registry [60], each with high response rates (>80%). Of the remaining studies, one recruited from two cancer registries but had a low response rate (<50%) [58], one comprised solely nurses [61], and the other included multiple cohorts with low response rates as well as the cohort of nurses [59]. All studies were observational so did not allocate participants to different treatments. It is possible that weight change around the time of diagnosis had an influence on which treatment participants received, so there was high risk of treatment allocation bias across all studies. The exposure (weight loss) was generally self-reported after the period of diagnosis and initial treatment. As such, data were likely to be selective for initial survivors (immortal time bias), as well as having the bias associated with self-report. The issue of early mortality was not addressed in the majority of studies, although one study attempted to collect follow-up data (weight one year prior to death) from a proxy where possible for participants who died between baseline and follow-up [57]. None of the studies examined the association between weight loss and selection for adjuvant treatment, but there were varying degrees of adjustment for treatment received; the risk of bias was therefore unclear. Data on adherence to treatment were not reported in any study, so the risk of adherence bias was unclear. Just one study reported associations between weight loss and both cancer-specific and all-cause mortality, with similar findings [57]; all other studies reported one mortality outcome so the risk of competing risk bias was unclear.

### Overview of study results

#### Overweight and obese cancer survivors

**Fig 3:**
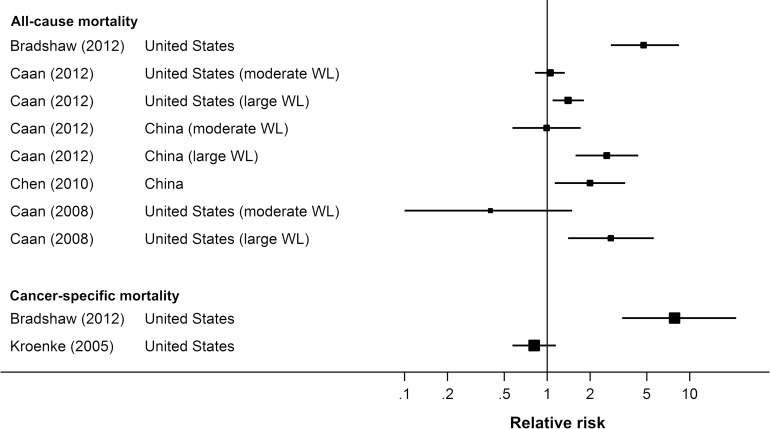
Forest plot of risk estimates from observational studies of weight loss and mortality outcomes in overweight and obese (BMI ≥25) breast cancer survivors.

**All-cause mortality:** All four studies that reported data on all-cause mortality found that, among overweight and obese breast cancer survivors, weight loss was associated with higher mortality compared with weight stability, with hazard ratios (HRs) ranging from 1.40 to 4.75 [57–60] (Fig 3).

All models adjusted for age at diagnosis, chemotherapy, and oestrogen and progesterone receptor status. Bradshaw *et al*. additionally adjusted for pre-diagnosis BMI, pre-diagnosis adult weight gain, and tumour size. Caan *et al*. (2008) additionally adjusted for stage, tamoxifen use, radiotherapy, number of positive nodes, smoking history, and physical activity, and Caan *et al*. (2012) adjusted for race, menopausal status, stage, positive nodes, radiotherapy, and smoking. Chen *et al*. adjusted for education, income, marital status, comorbidity, exercise participation, intake of meats, cruciferous vegetables, and soy protein, time interval from diagnosis to study enrolment, menopausal status, menopausal symptoms, type of surgery, radiotherapy, immunotherapy, tamoxifen use, and tumour-node metastasis stage.

In the two studies that subdivided participants who lost weight into ‘moderate’ and ‘large’ weight loss groups, only large weight loss (≥10% of initial weight) was significantly associated with increased risk of mortality; there was no significant difference in mortality risk between those who had a moderate weight loss (5–10% of initial weight) and those whose weight was stable [58,59].

The results in the Chinese cohort [59,60] were similar to those observed in the majority of the US cohorts, but more international studies are needed.

**Cancer-specific mortality:** Of the two studies that reported data on breast cancer-specific mortality, just one observed a significant association with weight loss. That study [57] found that overweight and obese women who lost weight were significantly *more* likely to die from breast cancer (HR 7.84, 95% CI 3.36–21.2). The other study [61] found no significant association between weight loss and breast cancer death (HR 0.81, 95% CI 0.57–1.15) (Fig 3).

Both studies adjusted for age and pre-diagnosis BMI, but Bradshaw *et al*. also adjusted for pre-diagnosis adult weight gain, hormone receptor status, and tumour size, while Kroenke *et al*. also adjusted for oral contraceptive use, parity and age at birth, menopausal status, age at menopause, use of hormone replacement therapy, protein intake, and tamoxifen use. Other notable differences between the studies were the study population and the definition of weight loss. Bradshaw *et al*.’s sample was recruited through hospitals after the point of diagnosis, whereas Kroenke *et al*.’s sample was a cohort of nurses recruited prior to diagnosis (the Nurses’ Health Study). However, it should be noted that the Nurses’ Health Study cohort was also included in the Breast Cancer Pooling Project which, consistent with Bradshaw *et al*.’s findings for all-cause mortality, found that weight loss was associated with higher all-cause mortality [59]. Weight loss was defined as a loss ≥5% of initial body weight in Bradshaw *et al*.’s study, and a BMI reduction of at least 0.5kg/m^2^ in Kroenke *et al*.’s study.

#### Non-overweight cancer survivors

**Fig 4:**
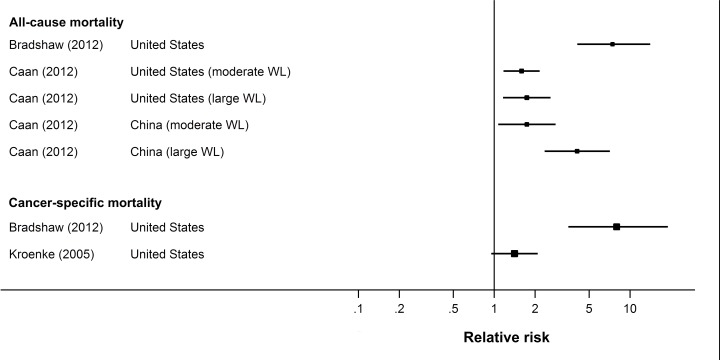
Forest plot of risk estimates from observational studies of weight loss and mortality outcomes in non-overweight (BMI <25) breast cancer survivors.

Of the five studies described above, four also presented data for non-overweight individuals (BMI <25). The results were the same as for the overweight and obese group. All three studies examining all-cause mortality found a higher risk for non-overweight cancer survivors who lost weight relative to those whose weight was stable, with HRs ranging from 1.59 to 7.43 [57,59,60]. Similarly, the results for breast cancer-specific mortality were mixed, with one study reporting increased risk among individuals with weight loss (HR 7.98, 95% CI 3.51–19.0) [57] and the other reporting no significant association with breast cancer mortality (HR 1.41, 95% CI 0.95–2.09) [61] (Fig 4).

In each study, the risk estimate associated with weight loss was somewhat higher for the non-overweight group than for the overweight and obese group. In addition, in contrast to the overweight and obese group, even moderate weight loss (5–10%) was associated with an increased all-cause mortality in non-overweight cancer survivors (USA: HR 1.59, 95% CI 1.17–2.16; China: HR 1.74, 95% CI 1.07–2.83) [59].

## Discussion

### Summary of findings

This systematic review examined the evidence from observational studies of overweight and obese cancer survivors that weight loss confers a mortality benefit. We found relevant literature to be scarce; just five studies met our inclusion criteria, all of which focused on breast cancer survivors. None of these provided evidence that weight loss improved survival for overweight and obese cancer survivors; rather, four studies found that weight loss was associated with increased mortality, and the fifth found no significant association with mortality. Similar results were observed in an additional study that examined weight loss and mortality in breast cancer survivors with a BMI ≥24 (and therefore did not meet our inclusion criteria), which reported a significantly higher risk of mortality with weight loss [62]. To help contextualise these findings, we also looked at the results for non-overweight cancer survivors where they were reported in the included studies. The results were largely similar, with all but one study finding weight loss to be associated with higher mortality and one finding no significant association; although effect sizes were slightly higher than for the overweight and obese survivors.

### Intentionality of weight loss: a fundamental issue

A crucial limitation of the studies included in our review is that none was able to distinguish between intentional and disease-related (unintentional) weight loss. Research in general population samples (not cancer survivors) indicates that unintentional weight loss is associated with increased risk of mortality whereas intentional weight loss has an overall neutral effect on survival [63]. In the context of breast cancer, both obesity and post-diagnostic weight gain have been shown to significantly increase the risk of mortality [15,64], and intentional weight loss in the general population has been shown to favourably affect numerous breast cancer-relevant risk factors and potential mediators, such as circulating oestrogens, sex-hormone binding globulin, inflammatory markers and insulin sensitivity [65], so one might expect to see a positive effect of intentional weight loss on survival.

Ideally, we would have limited the review to studies that looked at weight change entirely post-treatment to try to minimise the prevalence of unintentional weight loss resulting from disease or treatment, but we found none that met these criteria. Given the fact that several studies in this review did not control for tumour stage, patients may have had metastatic breast cancer at diagnosis and weight loss could have occurred as a result of their malignancy. Indeed, the strongest association between weight loss and mortality was seen in a study that did not adjust for disease stage [57]. Additionally, the majority of studies did not control for comorbidities, and cachexia occurs at later stages of many diseases, potentially confounding these observations. The inclusion of unintentional weight loss in their analyses is a potentially major source of bias which makes it impossible to draw conclusions regarding the safety of intentional weight loss for breast cancer survivors.

Analyses of survival outcomes by amount of weight loss are suggestive of unintentional weight loss driving significant associations with mortality. Large weight losses are unusual from traditional weight loss methods [66] and so are likely to be a sign of unintentional weight loss in this population group. In the two studies that examined mortality according to the amount of weight lost, it was only the large weight losses (≥10% of initial body weight) that were associated with increased mortality.

### Integration with the wider literature

The findings of the reviewed literature are seemingly at odds with evidence that weight loss in cancer survivors is associated with changes in biomarkers that are plausibly linked with cancer-related outcomes [28,30–33]. This is likely due to the findings of studies included in this review almost certainly being driven by unintentional weight loss. None of the studies relating weight loss to biomarker changes had mortality as an outcome in the original study, although one [28] observed lower mortality in the dietary intervention group (who also lost weight) as a post-hoc observation.

The majority of breast cancer survivors will die from causes other than breast cancer [67,68], with cardiovascular disease in particular accounting for a substantial proportion of deaths [67]. Breast cancer survivors have increased risk of cardiovascular disease and other competing sources of mortality such as second cancers [69,70], likely due to common risk factors such as obesity, as well as the impact of cancer treatments like anthracyclines and radiation. Weight loss has been shown to reduce the risk of these competing sources of mortality in other populations [71,72] and the same is likely true for women with breast cancer. Evidence for an association between post-diagnostic weight *gain* and increased risk of breast cancer recurrence and mortality [64] further call into question the biology which could underlie an association between weight loss and increased mortality risk.

Aside from effects on mortality, obesity has also been shown to increase the risk of morbidity in women with breast cancer. Surgical complications such as lymphedema, wound infections, poor wound healing, and unfavourable cosmetic outcomes with breast reconstruction are all more common in obese women [73–75]. Obesity is also associated with neuropathy as a result of chemotherapy, increased fatigue and reduced quality of life in women with breast cancer [76–81]. More than two dozen weight loss intervention studies have been performed in breast cancer populations, and these have shown that intentional weight loss leads to many improvements in symptoms and side effects of cancer therapy in breast cancer survivors [31,82–86]. These results indicate substantial benefits of weight loss for women with breast cancer. Notably, these studies have also shown no suggestion of increased risk of breast cancer recurrence or progression in women who lose weight.

### Sources of bias

In addition to the lack of consideration of weight loss intention, there were also other limitations and potential sources of bias in the extant literature. All the included studies focused on survivors of breast cancer, so results may not extend to male cancer survivors or female survivors of other cancers. In addition, the mean age of samples was relatively young at 54–59 years. The figures on breast cancer incidence in the UK indicate that around half of cases are diagnosed over the age of 60 and a quarter over the age of 75 [87], so these results may not generalise to older cancer survivors. The substantial overlap in study samples across four of the five studies we reviewed further limits the generalisability of findings, and may overinflate the current state of the evidence. It should also be noted that timespan for data collection in the included studies was from 1976 to 2008. Advances in treatment influencing survival may cause a time effect, and this time effect may vary across the included studies.

The cohorts were not specifically designed for the purpose of evaluating weight loss, so the analyses largely relied on self-reported data, and often with a recall component. While systematic under- or overestimation of weight should not substantially affect analyses of weight change, underreporting weight may have led to underestimation of effects in the non-overweight group through the inclusion of some overweight participants in this category. Likewise, the use of standard BMI cut-offs (as opposed to cut-offs adapted for Asian populations) to define overweight and obesity in the Chinese samples in two of the studies may also have resulted in effects in the non-overweight group being underestimated. In addition, the number of participants within the samples who lost weight was small, particularly in the ≥10% of baseline body weight bracket, making it difficult to derive reliable estimates of effects.

The studies did not consistently control for other important confounders of the association between weight loss and survival such as disease-related factors (e.g. stage, treatment, metastatic disease), health behaviours (e.g. physical activity, smoking), or comorbid health conditions, all of which may contribute to explaining differences in mortality between weight change groups. A number of the studies also failed to adjust for menopausal status despite significant associations with disease occurrence and recurrence and possible mortality [88,89].

Although all the studies stratified the results by weight status, none reported whether mean BMI was higher among overweight and obese weight losers than non-losers (the reference group). Given the evidence that being more overweight is associated with higher mortality [50,90], and that more overweight people are more likely to be trying to lose weight [10–14], if the group who lost weight were heavier at baseline, their risk would already be higher than the reference group, and therefore any risk reduction associated with weight loss would be attenuated. Only two studies attempted to control for this by adjusting for pre-diagnosis BMI.

The extent of potential bias in the studies we identified limits the insights they offer. Selection bias may be present in individual studies if the reasons for non-response are related to weight loss or mortality, although it is difficult to establish the degree to which any cumulative selection bias across studies influenced the results of this review. The results may also be biased by immortal time bias, because participants had to have survived for a certain period in order to have sufficient data for inclusion in the analyses. They could also be biased by between-group differences in allocation or adherence to treatment if this was influenced by weight change in the early post-diagnosis period. Most studies focused on all-cause mortality, precluding assessment of bias arising from non-cancer deaths.

### Future research

There are several ongoing randomised trials that will examine the impact of weight loss on breast cancer recurrence and survival. The Italian DIANA-5 trial [36] and the German SUCCESS-C trial [35] have completed recruitment, and in North America the ENERGY trial is currently recruiting [39] and the BWEL trial is starting recruitment in August 2016 [40]. These trials will provide conclusive evidence on the impact of weight loss on breast cancer outcomes, but further research is needed to extend the evidence base to other cancer sites.

In observational studies, it would be valuable to have better assessments of weight loss as an exposure, with a move toward objective measurements of body weight. Some assessment of intentionality is also needed. Defining intentionality is not easy, and will likely require more than simply asking participants whether or not they were trying to lose weight. Weight loss attempts are reported by a large majority of overweight and obese individuals [10–13], and so they may misattribute unintentional weight loss to their own efforts. Studies that focus on the period of time after treatment has been completed and weight has returned to normal could help to limit the complication of unintentional weight loss for observational research in this area. Future studies also need to take account of treatment allocation and adherence; both of which might be influenced by weight status and weight change.

### Limitations of the present review

This systematic review has several limitations. It was up-to-date at the time of submission, but as this topic is one of growing interest, further relevant studies may have since been published. We did not assess the likelihood of publication bias, and it is possible that analyses that found a neutral effect of weight loss on mortality did not make it to publication. Many systematic reviews use meta-analysis to increase the statistical power to examine whether or not an association exists across published research results. We concluded that this was inappropriate in the present review because of the small number of studies and their variation in relation to the timing and definition of weight loss, adjustment for potential confounders, and overlap in the study populations. However, as the literature on this topic grows, such a meta-analysis may become possible.

### Conclusions

We were unable to identify any evidence on weight loss and mortality in non-breast cancer populations. It is difficult to draw conclusions about the relationship between weight loss and breast cancer outcomes from the limited observational data available, especially in terms of breast cancer mortality. Ongoing definite trials testing the impact of purposeful weight loss on breast cancer prognosis are important first steps for establishing whether weight loss confers a mortality advantage for overweight and obese cancer survivors. However, trials with survivors of other cancer types, and observational studies with improved assessments of weight, weight loss intentionality and which take into account important confounders are also needed.

## Supporting Information

S1 Table PRISMA checklist. (DOC)

S2 Table Quality assessment tool. (PPTX)
