# Supplementary material for: Weight Loss and Mortality in Overweight and Obese Cancer Survivors: A Systematic Review
Source: PLoS One. 2017 Jan 6;12(1):e0169173. doi: 10.1371/journal.pone.0169173 (PMC5218508; doi:10.1371/journal.pone.0169173)
Supplement: S2 Table — (PPTX) [file pone.0169173.s002.pptx]

## Slide 1
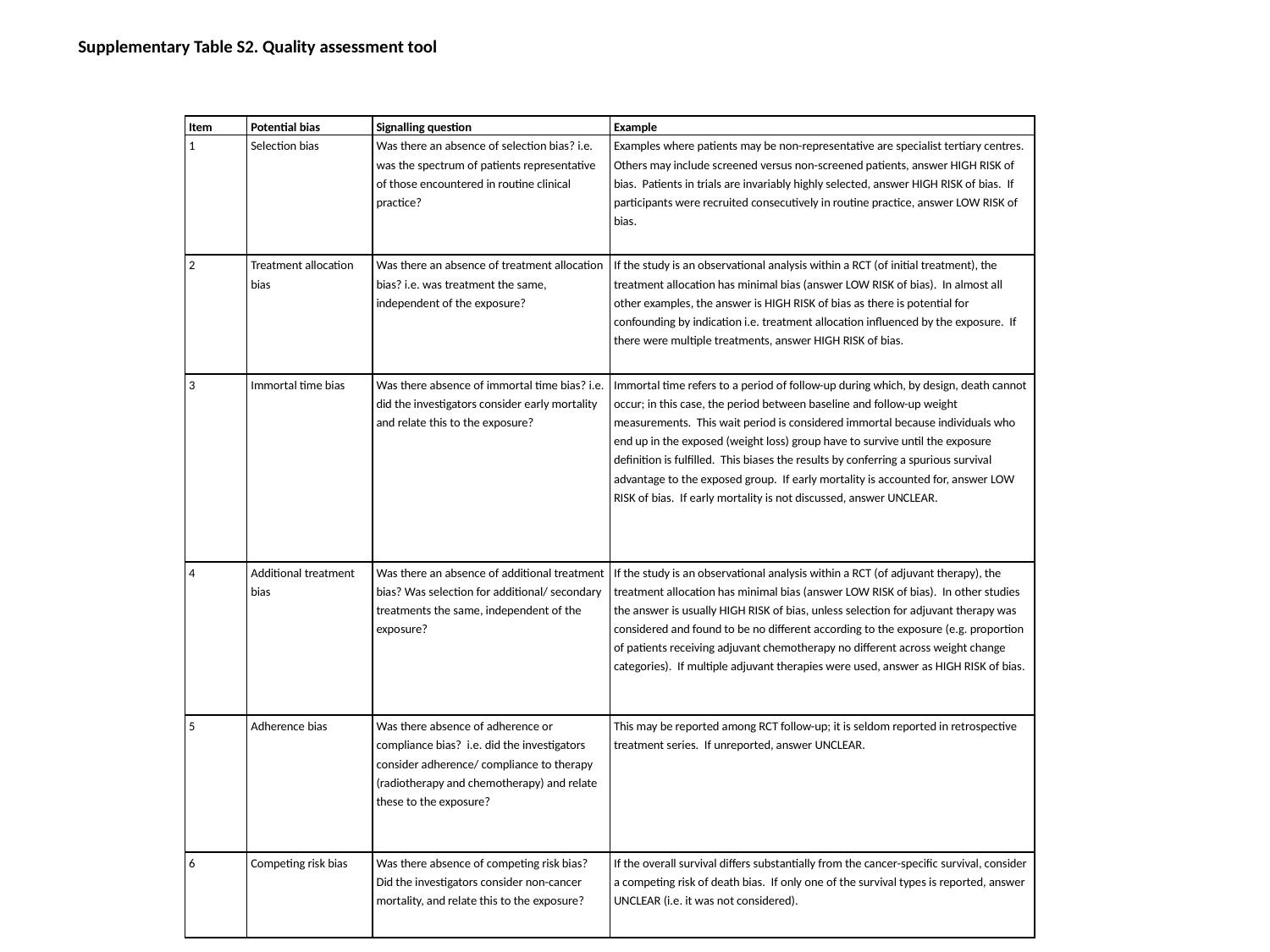

Supplementary Table S2. Quality assessment tool
| Item | Potential bias | Signalling question | Example |
| --- | --- | --- | --- |
| 1 | Selection bias | Was there an absence of selection bias? i.e. was the spectrum of patients representative of those encountered in routine clinical practice? | Examples where patients may be non-representative are specialist tertiary centres. Others may include screened versus non-screened patients, answer HIGH RISK of bias. Patients in trials are invariably highly selected, answer HIGH RISK of bias. If participants were recruited consecutively in routine practice, answer LOW RISK of bias. |
| 2 | Treatment allocation bias | Was there an absence of treatment allocation bias? i.e. was treatment the same, independent of the exposure? | If the study is an observational analysis within a RCT (of initial treatment), the treatment allocation has minimal bias (answer LOW RISK of bias). In almost all other examples, the answer is HIGH RISK of bias as there is potential for confounding by indication i.e. treatment allocation influenced by the exposure. If there were multiple treatments, answer HIGH RISK of bias. |
| 3 | Immortal time bias | Was there absence of immortal time bias? i.e. did the investigators consider early mortality and relate this to the exposure? | Immortal time refers to a period of follow-up during which, by design, death cannot occur; in this case, the period between baseline and follow-up weight measurements. This wait period is considered immortal because individuals who end up in the exposed (weight loss) group have to survive until the exposure definition is fulfilled. This biases the results by conferring a spurious survival advantage to the exposed group. If early mortality is accounted for, answer LOW RISK of bias. If early mortality is not discussed, answer UNCLEAR. |
| 4 | Additional treatment bias | Was there an absence of additional treatment bias? Was selection for additional/ secondary treatments the same, independent of the exposure? | If the study is an observational analysis within a RCT (of adjuvant therapy), the treatment allocation has minimal bias (answer LOW RISK of bias). In other studies the answer is usually HIGH RISK of bias, unless selection for adjuvant therapy was considered and found to be no different according to the exposure (e.g. proportion of patients receiving adjuvant chemotherapy no different across weight change categories). If multiple adjuvant therapies were used, answer as HIGH RISK of bias. |
| 5 | Adherence bias | Was there absence of adherence or compliance bias? i.e. did the investigators consider adherence/ compliance to therapy (radiotherapy and chemotherapy) and relate these to the exposure? | This may be reported among RCT follow-up; it is seldom reported in retrospective treatment series. If unreported, answer UNCLEAR. |
| 6 | Competing risk bias | Was there absence of competing risk bias? Did the investigators consider non-cancer mortality, and relate this to the exposure? | If the overall survival differs substantially from the cancer-specific survival, consider a competing risk of death bias. If only one of the survival types is reported, answer UNCLEAR (i.e. it was not considered). |
